# Allicin Could Potentially Alleviate Oral Cancer Pain by Inhibiting “Pain Mediators” TNF-alpha, IL-8, and Endothelin

**DOI:** 10.3390/cimb43010016

**Published:** 2021-05-23

**Authors:** Abdulwahab H. Alamir, Shankargouda Patil

**Affiliations:** 1 Division of Oral Medicine, Department of Maxillofacial Surgery and Diagnostic Sciences, College of Dentistry, Jazan University, Jazan 45142, Saudi Arabia; dr.abdulwahab@hotmail.com; 2 Division of Oral Pathology, Department of Maxillofacial Surgery and Diagnostic Sciences, College of Dentistry, Jazan University, Jazan 45142, Saudi Arabia

**Keywords:** allicin, cancer stem cells, oral cancer, pain

## Abstract

To evaluate the effects of allicin on mediators of pain secreted by oral cancer cells in vitro, single-cell suspensions were prepared by enzymatic method from oral squamous cell carcinoma (OSCC). Cancer stem cells were isolated by the CD133+ selection method with magnetic cell sorting. Stemness markers were checked in both cancer cells and cancer stem cells by RT-PCR. Comparative analysis of pain mediators TNF-alpha, IL-8, and endothelin at both RNA and protein levels for normal epithelial cells, cancer cells, and cancer stem cells was carried out with and without allicin treatment. CD133 and CD44 expression levels were checked in cancer cells and cancer stem cells flow cytometrically. Allicin inhibited both gene and protein expression of TNF-alpha, IL-8, and endothelin in both cancer cells and cancer stem cells. Allicin is more likely to be a promising treatment in alleviating the levels of pain and inflammation in OSCCs.

## 1. Introduction

Pain is a common symptom of most progressive diseases, including cancer. Pain noted in cancer patients could be a result of the tumor impinging on a nerve or due to active secretion of nociceptor mediators by the tumor cells or due to the associated inflammation [1]. In oral cancer, pain is largely attributed to the nociceptor mediators including tumor necrosis factor-γ, proteases, and nerve growth factor [2,3,4,5]. Unlike gastrointestinal tract or pelvic cancer, the pain in oral cancer is highly localized due to the dense oral innervation of the trigeminal nerve [6]. Also, the constant stimulations exerted on the oral cavity in the form of speech, mastication, and deglutition aggravates the oral cancer pain [7]. Thus, most oral cancer pain is attributed as function related rather than spontaneous pain. Studies have also explored the association between oral cancer pain and disease progression. Nodal metastasis has shown to be associated with higher pain-induced functional restrictions, indicating a positive correlation between the intensity of oral cancer pain and cancer progression [1,4,8]. Furthermore, surgical excision of the tumor has shown to dramatically reduce the associated pain, providing further evidence as to the direct association between the pain and cancer [1,2,4,9]. Epigenetic mechanisms, especially, micro-RNA (miR-125b, miR-181, and miR-339) have shown to be a major factor in oral cancer pain, mediating several pathways culminating in central sensitization, wherein even a non-nociceptive stimulus could trigger a nociceptive response [6,10]. Although there are several therapeutic regimens available to control oral cancer pain, the higher dosages of drugs given to counter oral cancer pain often lead to respiratory depression, constipation, nausea, and vomiting. Thus, the medications provided to relieve the oral cancer pain, in turn, reduce the overall quality of life, predisposing the patients towards progressive depression [11,12].

Cancer-associated pain is an ever-growing public health concern. Oral cancer pain and the underlying mechanisms of the mediators of this pain are poorly understood to date. With the recent treatment advances, patients with oral cancer are surviving longer but the pain due to the inflammatory mediators brings about a poor quality of life. Oral cancer pain significantly affects most of the functions which are routinely necessary such as talking and swallowing [1,11,13]. Therefore, we hypothesize that the biologic mechanisms of oral cancer pain would be associated within the tumor microenvironment and studying which would further help to understand the crosstalk between tumor cells and healthy tissues.

In this study, we are mainly focusing on largely associated oral cancer pain mediators TNF-alpha, IL-8, and endothelin secreted by oral tumor cells and cancer stem cells and the effect of allicin in reducing these pain mediators.

## 2. Materials and Methods

### 2.1. Sample Collection and Ethical Permissions

Scientific research (IRB)-College of Dentistry, Jazan University approved the study (reference no. CODJU-19237). Samples were collected after obtaining patient informed consent. The samples were collected in a sterile container containing complete media and transported directly to the laboratory.

### 2.2. Preparation of the Single-Cell Suspension

Biopsy samples were obtained from histopathologically normal oral mucosa during the surgical extraction of third molars of the subjects and primary oral tumors from oral squamous cell carcinoma (OSCC) patients were collected. The tissues were rinsed thoroughly with sterile phosphate-buffered saline (PBS) containing antibiotic antimycotic solution. Furthermore, the tissues were minced with a sterile blade and then directly subjected to enzymatic digestion with an enzyme cocktail of 0.4% collagenase II and 0.2 mg/mL dispase. The minced tissue was incubated in the enzyme cocktail for an hour. After neutralizing the enzyme action with fetal bovine serum, the digested tissue was passed through a sterile cell strainer of suitable pore size. Furthermore, the centrifugation was done at 2000 rpm for 5 min. The pellet obtained was rinsed twice with complete media. Finally, the pellet was split into two parts to seed directly in the culture flask and to sort CD133+ cells.

### 2.3. Treatment Groups

For MTT assay, RT-PCR, and ELISA assay, allicin at 0 ng/mL (control), 20 ng/mL, 50 ng/mL, and 100 ng/mL was used to treat OSCC cells. The treatment was given for 48 h with a complete growth medium.

### 2.4. 3-(4,5-Dimethylthiazol-2-yl)-2,5-Diphenyltetrazolium Bromide (MTT) Assay

Cell proliferation was determined by using an MTT assay. The cells were seeded into 96-well plates and incubated with media and appropriate treatment for 48 h. Following this, 0.5 mg/mL MTT solution was added to each well, and the plates were incubated for 4 h at 37 °C. Subsequently, the media was replaced with 100 µL dimethyl sulfoxide (DMSO) in every well. The absorbance was taken at 570 nm using a spectrophotometer.

### 2.5. Magnetic Sorting of CD133+ Cells

Total cancer cells were subjected to magnetic cell sorting by using Miltenyi CD133 MicroBead Kit for human tumor tissue according to the protocol provided with the kit. The sorted CD133+ cells were acquired on a flow cytometer to check the purity and then seeded into a culture flask for further expansion.

### 2.6. RT-PCR for Stemness Markers and Gene Expression Analysis of TNF-alpha, IL-8, and Endothelin

The treated and untreated cells with allicin (Purchased from Santacruz Biotechnology with more than 98 percent purity, CAS 539-86-6) were pelleted down and by the Trizol method total RNA was isolated. Reverse transcription was done of 1 μg total RNA using a cDNA synthesis kit according to the given guidelines. Quantitative gene expression analysis for TNF-alpha, IL-8, and endothelin was carried out using SYBR Green PCR chemistry master mix on QuantStudio 5 Real-Time PCR machine. Expressions of target genes were normalized to ß-actin using the ΔΔCt method. The cycle threshold (CT) values for each gene were corrected using the mean CT value. RT-PCR data were quantified using the 2^−ΔΔCt^ method and presented as relative gene expression normalized to the average CT for the β-actin gene. The primers used are listed in Table 1.

### 2.7. ELISA for Analysis of Protein Levels of TNF-alpha, IL-8, and Endothelin

The analysis of protein levels of pain mediators was carried out by using KRIBIOLISA human ELISA kits according to the manufacturer’s guidelines. Briefly, 100 μL/well samples and standards were added to the plate and two-fold serial dilutions of top standard. The plate was incubated at room temperature for 2 h. After incubation, the plate was washed four times. After adding 100 μL detection antibody to each well, the plate was further incubated for 2 h at room temperature. Again, the plate was washed four times. 100 μL of diluted streptavidin-HRP was added to each well and incubated for 30 min at room temperature. After washing the plate four times, 100 μL of TMB substrate was added and incubated for 30 min in the dark. The reaction was stopped by the addition of 100 μL stop solution. The absorbance was read at 450 nm on a spectrophotometer.

### 2.8. Statistical Analysis

The experiment was repeated three times and all the samples were run in triplicates (n = 10). All the values in the data were presented as mean ± standard deviation. Experimental sets were compared using ANOVA as the statistical test. For all analyses *p*-value < 0.05 was considered significant (levels of significance: * *p* < 0.05 and ** *p* < 0.01).

## 3. Results

### 3.1. Sorted CD133+ Cells Show High Expression of Stemness and Pluripotency Markers

**Figure 1:**
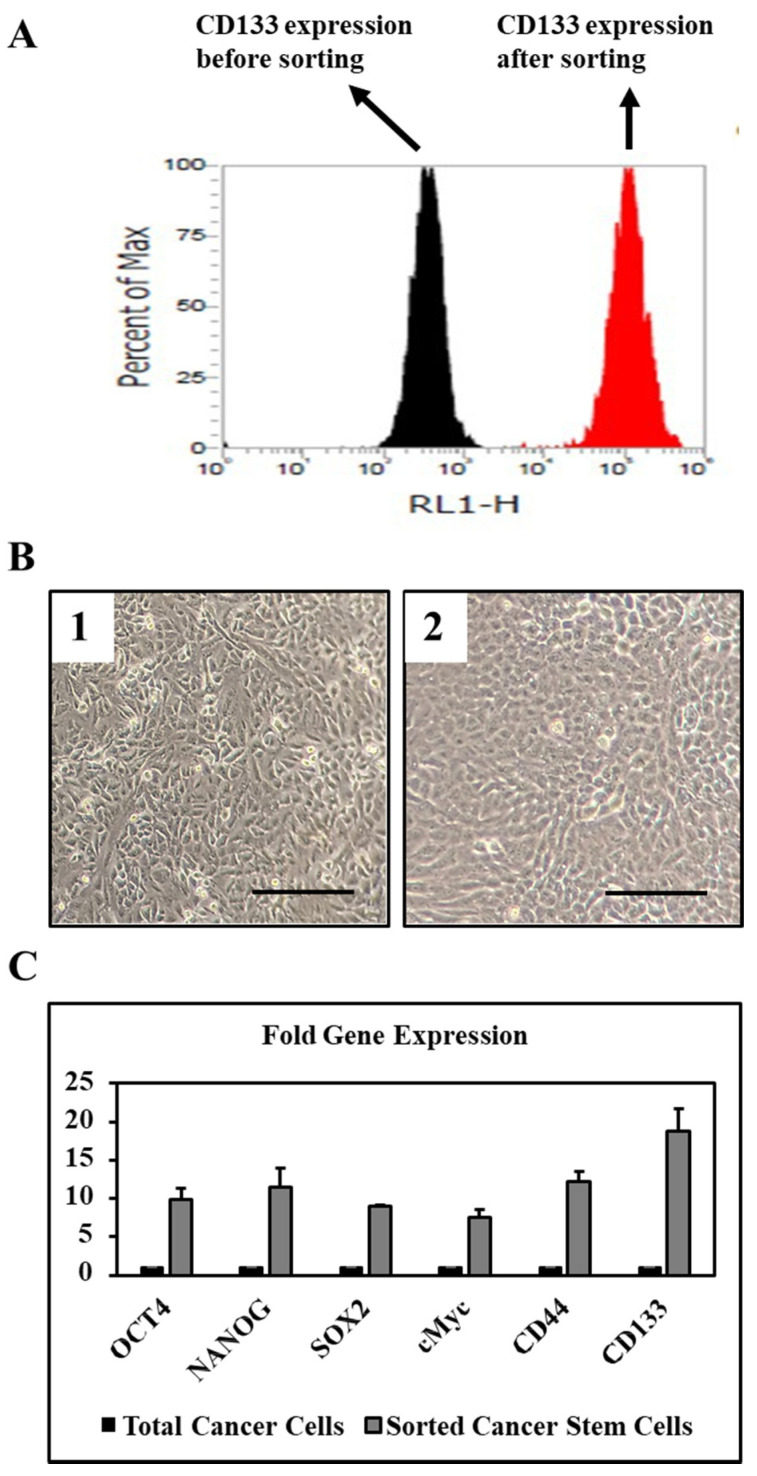
OSCC tissues were digested enzymatically to prepare single cell suspensions and further used for experiments and culture. (**A**) CD133 expression in magnetically sorted oral cancer stem cells. (**B**) Morphology of total cancer cell population B1 and CD133+ sorted cancer stem cells B2. Scale bar = 100 μm. (**C**) Fold gene expression of stemness and pluripotency related genes in CD133+ sorted cancer stem cells.

Sorted CD133+ cells from total OSCC cells showed very high expression of stemness and pluripotency related genes at the basal level (Figure 1A–C). Also, gene expression of cancer stem cell-related surface markers CD44 and CD133 were found to be highly expressed in the sorted CD133+ cells at the basal level (Figure 1A–C).

### 3.2. High Concentration of Allicin Does Not Affect Cell Viability Significantly

**Figure 2:**
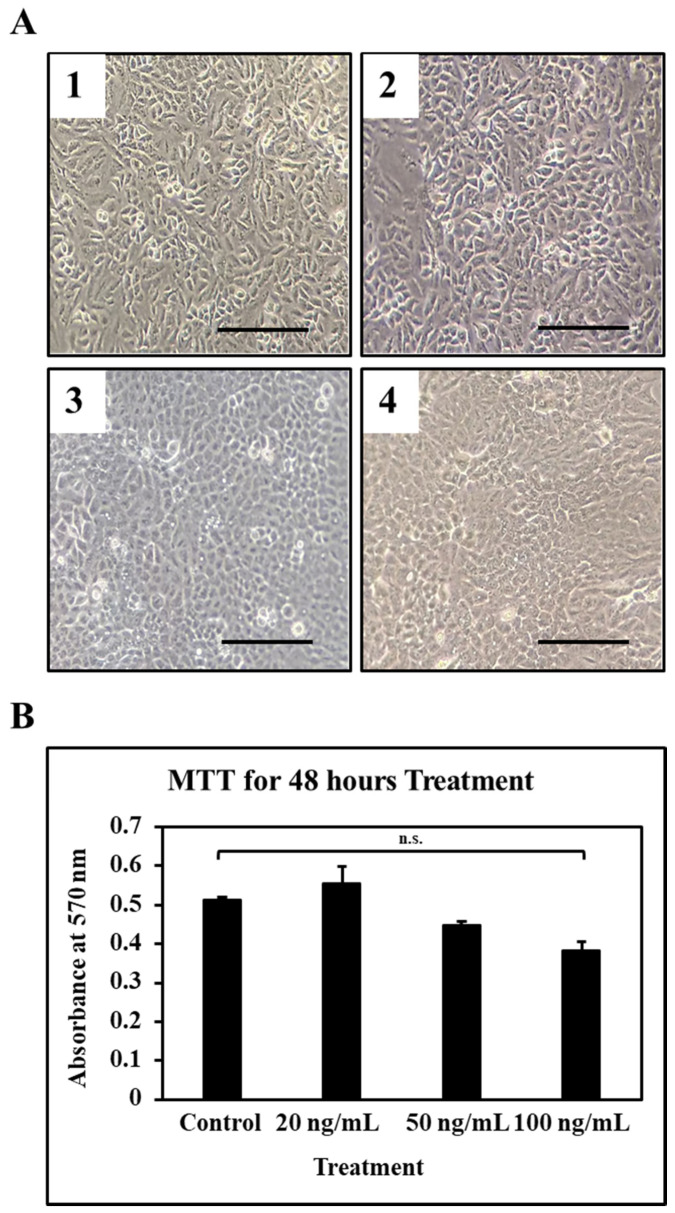
OSCC cells were treated with different concentrations of allicin and MTT assay was performed to check the cell viability. (**A**) Morphology of the OSCC cells after treatment with allicin: Control A1, 20 ng/mL A2, 50 ng/mL A3, 100 ng/mL A4. Scale bar = 100 μm. (**B**) MTT assay. n.s. not significant.

Lower concentrations of allicin were found to be safe in terms of cell death as the MTT results showed not a significant reduction in cellular viability (Figure 2A,B). Amongst which the higher concentration which is 100 ng/mL was found to be affecting the cell viability (Figure 2A,B).

### 3.3. Allicin Downregulates Gene Expression of Pain Mediators in a Dose-Dependent Manner

**Figure 3:**
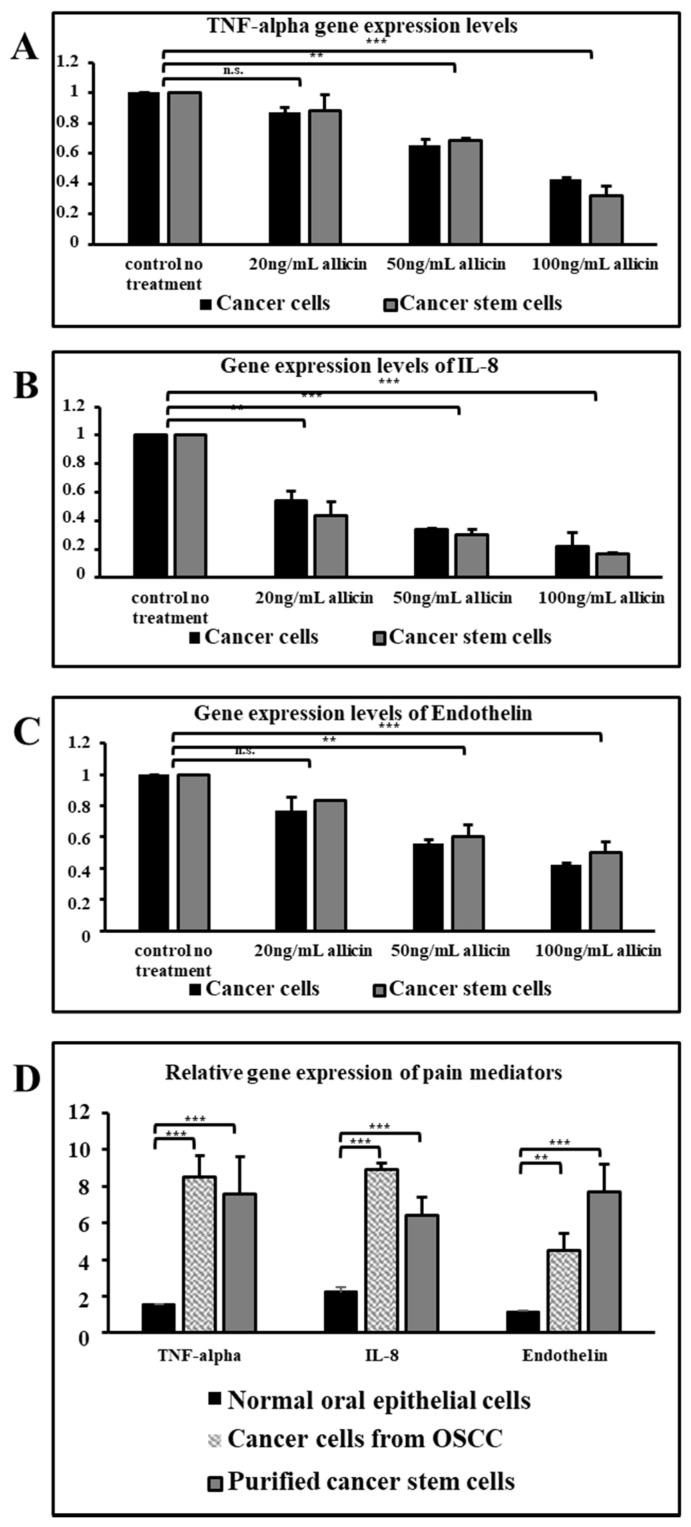
Real time PCR was performed to assess the gene expressions of pain mediators TNF-α, IL-8, and endothelin. (**A**) TNF-α gene expression levels. (**B**) IL-8 gene expression levels. (**C**) Endothelin gene expression levels. (**D**) Relative gene expressions of pain mediators. n.s. not significant, ** *p* < 0.05, *** *p* < 0.01.

Lower concentrations of allicin did not affect the gene expression of TNF-alpha and endothelin (Figure 3A,C) but all three concentrations of allicin were found to be affecting the gene expression of IL-8 significantly in both cancer cells and sorted cancer stem cells (Figure 3B,D).

### 3.4. Allicin Inhibits the Secretion of Pain Mediators at the Protein Level in a Dose-Dependent Manner

**Figure 4:**
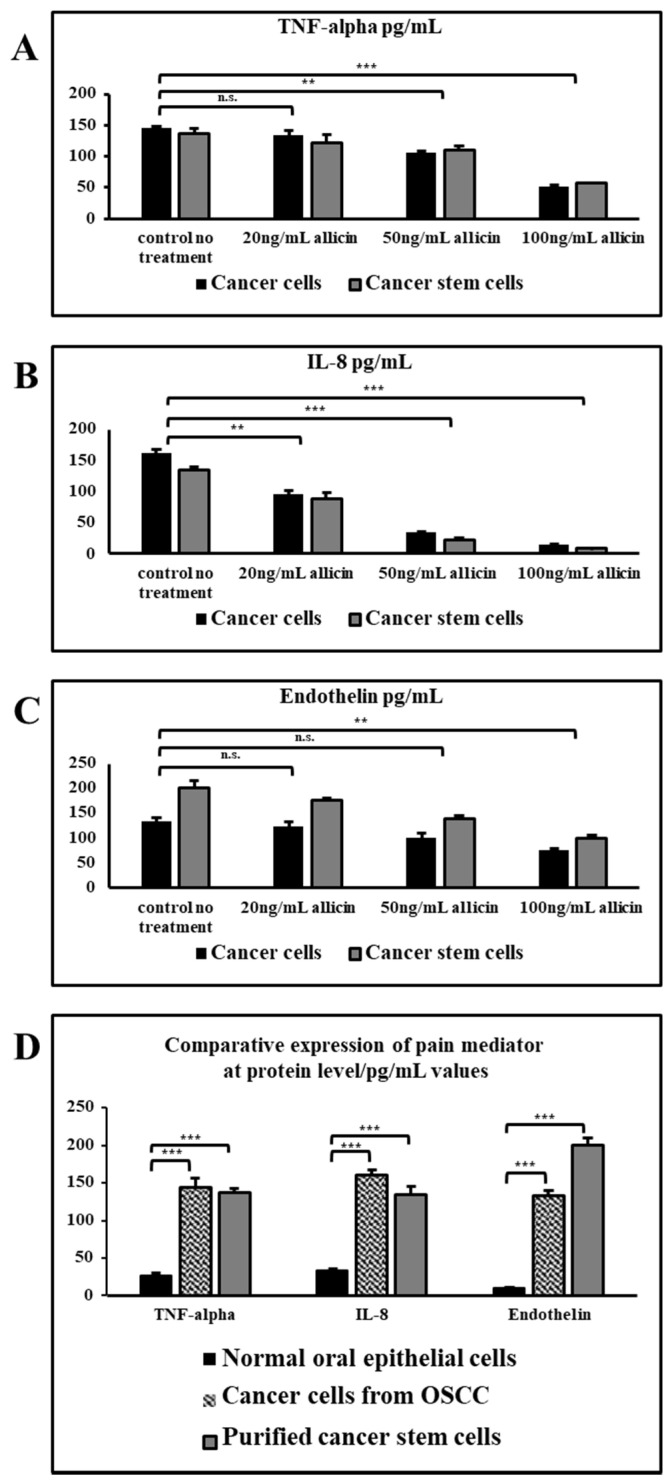
ELISA was performed to measure the secretion levels in pg/mL of pain mediators TNF-α, IL-8, and endothelin. (**A**) Protein levels of TNF-α. (**B**) Protein levels of IL-8. (**C**) Protein levels of Endothelin. (**D**) Comparative expressions of pain mediators at protein level in pg/mL values. n.s. not significant, ** *p* < 0.05, *** *p* < 0.01.

A higher concentration of allicin significantly reduced the secreted protein levels of all the three pain mediators TNF-alpha, IL-8, and endothelin (Figure 4A–C). IL-8 secretion was significantly reduced in all three concentrations of allicin in both cancer cells and cancer stem cells (Figure 4D).

### 3.5. Allicin Significantly Reduces the Expression of Cancer Stem Cell Markers CD44 and CD133 in a Dose-Dependent Manner

**Figure 5:**
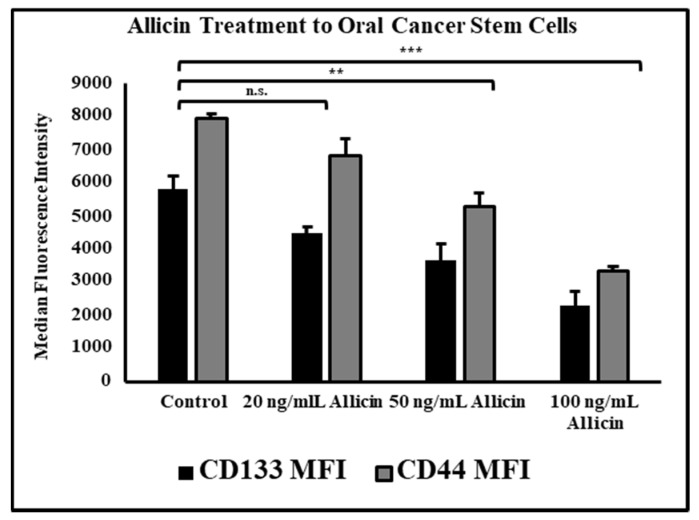
OSCC stem cells were treated with the different concentrations of allicin and acquired on flow cytometer to examine the expression of cancer stem cell markers CD133 and CD44. n.s. not significant, ** *p* < 0.05, *** *p* < 0.01.

Allicin treatment at higher concentrations of 50 ng/mL and 100 ng/mL significantly reduced expression of cancer stem cell surface markers CD44 and CD133 measured as median fluorescence intensity on a flow cytometer (Figure 5).

## 4. Discussion

Pain in oral cancer is often considered to have originated from the tumor cells rather than the size of the tumor [3,4,5,6]. There are several reports on various oral cancer cell lines and animal models that clearly state the pain is caused by inflammatory cytokines secreted by the OSCC cells [2,5,6,7,8]. Still, there is no further proof that exactly which secretory factors are responsible to cause such debilitating pain. Some reports show a putative cell population within a tumor that could be responsible for the production of the pain mediators [3,9,10,11,12,13]. Therefore, we suspect that it could be the cancer stem cell (CSCs) population that perform a constellation set of functions for the growth of the tumor. Allicin has been reported to have the pain-reducing ability in animal models of pain. In cancer, allicin has been shown to reduce inflammation caused by inflammatory cytokines [14,15]. Here we have attempted to study some molecules implicated in the initiation of pain due to tumor formation and the effect of allicin treatment on these pain mediators and the stemness markers.

After isolating the CD133+ CSCs from OSCC tissue cells, we found that the stemness and pluripotency related genes such as OCT4, SOX2, and NANOG were expressed at the basal level. Also, there was a very high expression of the CD133 gene at the basal level in CSCs. The CSCs need the expression of these genes to maintain stemness and to give rise to other cell types that perform a vital role in the growth of the tumor. Therefore, this data is a very important tool to know the potential of the tumor.

TNF-alpha is a pro-inflammatory cytokine and IL-8 is a potential chemo-attractant that is responsible to attract many immune cells to the site of inflammation which further progresses into pain [16,17,18]. Whereas endothelin-1 is the molecule that acts directly on the nociceptors and further enhances the effect of other pain-inducing molecules [19]. We wanted to check the relationship in these three molecules concerning the pain-inducing genetic cascade. With the allicin treatment, it was evident that the expression of all these three molecules was strikingly reduced in a dose-dependent manner at higher doses. Therefore, it was also observed that the effect of allicin was not much different in both total OSCC cells and CSCs at gene expression level and protein level. Though the lower concentration of allicin did not show much difference in downregulation of TNF-alpha and ET-1 gene expression, the expression of IL-8 was significantly reduced by the lower dose of allicin. The same trend was observed at the protein level also. The secreted molecules were found to be decreasing at the higher concentrations of allicin treatment. The relative expression of these proteins was extraordinarily higher when compared with the normal oral epithelial cells.

The microenvironment in the OSCC tissues has an augmented ET-1 level and has reported mechanical stimulation-induced functional pain. To simulate OSCC associated mechanical allodynia, Pickering et al. formulated an in-vivo model, wherein the mouse hind paw was inoculated with OSCC cells. The animal model confirmed ET-1 role in OSCC associated pain. It was shown that cancer pain was largely attributed to the ET-1 concentration rather than the tumor volume [10]. In-vivo animal model studies have shown the pain induced through proinflammatory cytokines (TNF-a and IL-1b) administration could be alleviated with anti-inflammatory cytokines/inhibitors of proinflammatory cytokine. Cytokines are major mediators for cancer pain in humans. Loss of balance in the proinflammatory and anti-inflammatory cytokine levels in fluids such as blood, cerebrospinal fluid, and tissues such as skin and nerve are attributed as the major cause for pain. In conditions such as painful neuropathy, an augmented pro-inflammatory (IL-2 and TNF-alpha) mRNA levels and attenuated anti-inflammatory (IL-4 and IL-10) mRNA levels were noted. Monocyte/macrophage released cytokines (IL-6, IL-1b, and TNF-a) cause hepatocyte activation inducing hepatic acute phase response, culminating serum proteins (CRP) synthesis [16].

Garlic’s therapeutic properties are largely attributed to the allicin-based organosulfur compounds (OSCs). Allinase enzyme produces allicin from alliin, which in turn is further processed. The major component of a plant’s defense system is allinases, which are released when there is damage to the plant. Cell-growth stimulatory proteins are inhibited by OSCs derived from garlic thereby attenuating most of the Hanahan and Weinberg defined cancer hallmarks.

Cellular redox systems including cysteinyl S-conjugates activation through β-lyase reactions and production of reactive persulfide or sulfane sulfur progenitors are affected by the OSCs. These components may in turn induce reaction with cysteine moieties on redox-sensitive proteins. Garlic-derived S-allylcysteine inhibits prostate cancer cells. Allicin has caused apoptosis (both caspase-dependent and independent) in several types of cancer cells. It has also been shown to alleviate chemotherapeutics-associated toxicity. Allicin degradation products such as diallyl sulfides induce anti-cancer activities by augmenting apoptosis and cell cycle arrest [14]. Allicin potentially acted on the inflammatory mediators, but our question was whether the stemness of CSCs in OSCC tissue is affecting the expression of these molecules, and allicin treatment is acting upon the stemness markers CD133 and CD44 of CSCs. Therefore, we treated the CSCs with the same concentrations of allicin and acquired the cells on the flow cytometer. Remarkably, the expressions of CSC surface molecules were found to be decreasing in the allicin-treated CSCs in the same trend as the inflammatory mediators. This data signifies the role of stemness in inflammation-mediated pain.

However, this is a very preliminary study showing inflammatory cytokines acting as pain mediators in oral cancer. A qualitative study is required on a significant number of samples with sophisticated experimental techniques and high-throughput data analysis. Our future studies will be focusing on a wide array of upstream and downstream regulators of these pain mediators.

## 5. Conclusions

Though we were able to study the effect of allicin on the primary OSCC tumor cells and tumor stem cells, further investigations are needed at both in vitro and in vivo levels to correlate these complex molecular interactions to understand the root cause of pain to develop better therapeutic strategies. In conclusion, allicin can be utilized along with a routine pain management system for cancer-related pain in the oral cavity.

## Figures and Tables

**Table 1:** List of primers.

Gene	Forward Primer	Reverse Primer
TNF-alpha	5′-CCG ATG GGT TGT ACC TTG TC-3′	5′-GGGCTG GGT AGA GAA TGG AT-3′
IL-8	5′-GTG CAG TTT TGC CAA GGA GT-3′	5′-TTA TGA ATT CTC AGC CCT CTT CAA-3′
Endothelin 1 (ET-1)	5′-CTT TGA GGG ACC TGA AGC TG-3′	5′-AGT TCT TTT CCT GCT TGG CA-3′
